# Supplementary material for: Identification of Factors Affecting the Increased Percentage of CGA Recommendations among Patients on Geriatric Ward
Source: Int J Environ Res Public Health. 2023 Jan 23;20(3):2065. doi: 10.3390/ijerph20032065 (PMC9915924; doi:10.3390/ijerph20032065)
Supplement: Supplementary file 1 [file ijerph-20-02065-s001.zip › Figure S1.pdf]

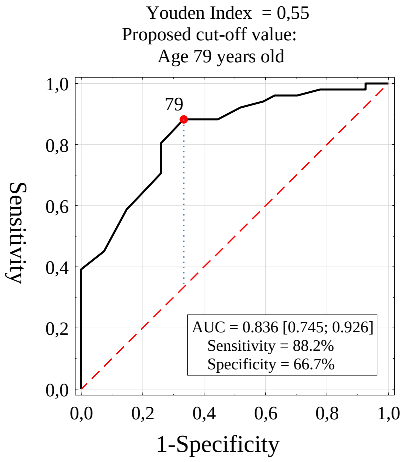

Figure S1. ROC curve to estimate the probability of a recommendation for a CGA based on the patient's age, area under the curve (AUC), and sensitivity and specificity for the proposed cut-off value
