# Supplementary material for: Identification of Factors Affecting the Increased Percentage of CGA Recommendations among Patients on Geriatric Ward
Source: Int J Environ Res Public Health. 2023 Jan 23;20(3):2065. doi: 10.3390/ijerph20032065 (PMC9915924; doi:10.3390/ijerph20032065)
Supplement: Supplementary file 1 [file ijerph-20-02065-s001.zip › Figure S2.pdf]

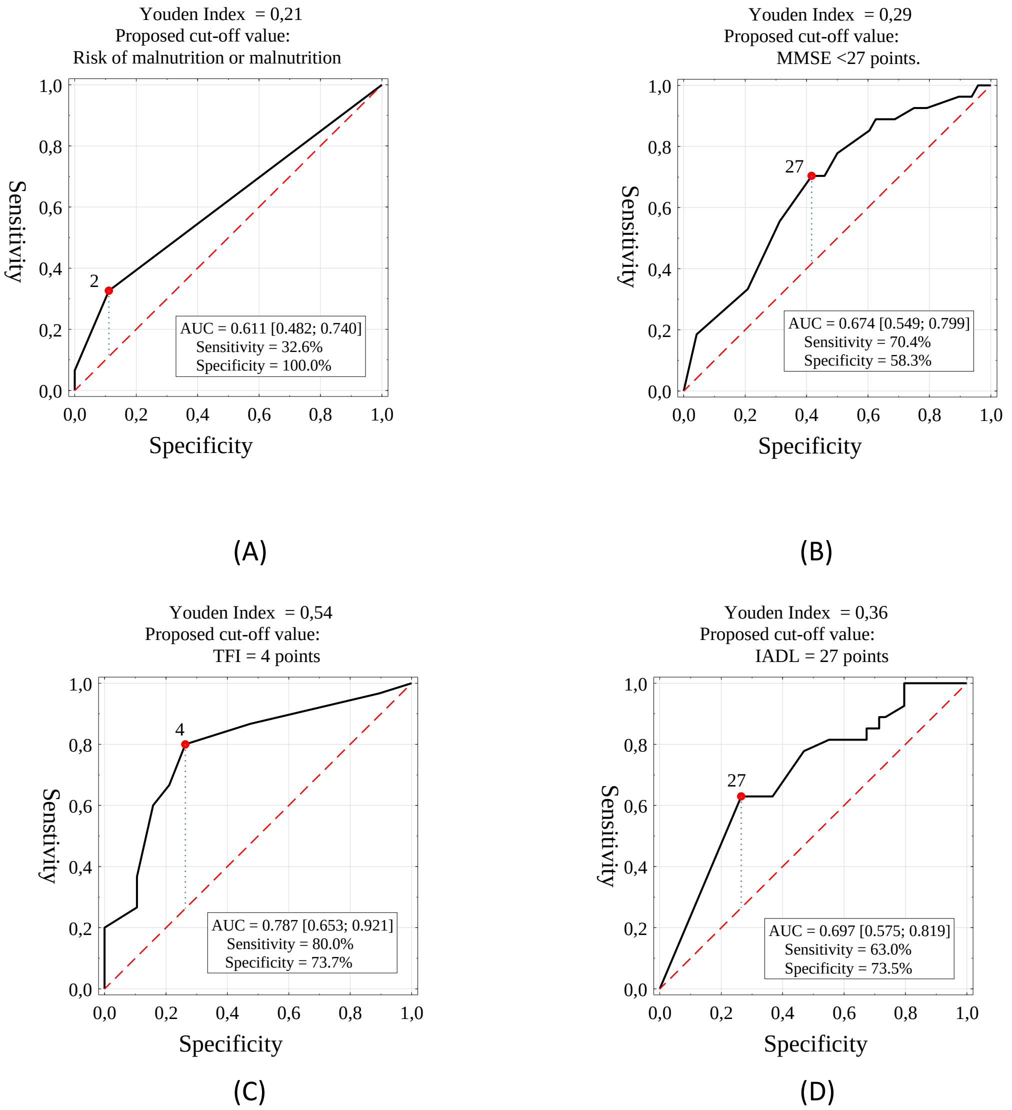

Figure S2. ROC curve for estimating the probability of a recommendation for COG based on A) nutritional status, B) mental state, C) level of fragility syndrome and D) the patient's ability to perform complex life activities; area under the curve (AUC) and sensitivity and specificity for the proposed cut-off values
